# Occult Hepatitis C Virus Infection in Candidates for Liver Transplant With Cryptogenic Cirrhosis

**DOI:** 10.5812/hepatmon.11290

**Published:** 2013-08-05

**Authors:** Hossein Keyvani, Farah Bokharaei-Salim, Seyed Hamidreza Monavari, Maryam Esghaei, Mohssen Nassiri Toosi, Shahin Fakhim, Zohreh Azita Sadigh, Seyed Moayed Alavian

**Affiliations:** 1 Department of Virology, Iran University of Medical Sciences, Tehran, IR Iran; 2 Department of Virology and Anti-Microbial Resistant Research Center, Iran University of Medical Sciences, Tehran, IR Iran; 3 Department of Gastroenterology, Imam Khomeini Hospital, Tehran University of Medical Sciences, Tehran, IR Iran; 4 Department of Civil Engineering, Islamic Azad University, Shahre Qods, Tehran, IR Iran; 5 Razi Vaccine and Serum Research Institute, Karaj, IR Iran; 6 Baqiyatallah Research Center for Gastroenterology and Liver Diseases, Tehran, IR Iran

**Keywords:** Hepatitis C Virus, Occult Infection, Peripheral Blood Mononuclear Cells, Cryptogenic, Cirrhosis, Liver Transplantation

## Abstract

**Background:**

Occult hepatitis C virus (HCV) infection is a new entity described by the presence of HCV-RNA in liver biopsy and/or peripheral blood mononuclear cell (PBMC) specimens, and undetectable levels or absence of HCV-RNA and in the absence or presence of anti HCV antibodies in plasma by current laboratory methods.

**Objectives:**

To evaluate the detection of HCV-RNA in PBMC specimens of the liver transplant candidates with cryptogenic cirrhosis by reverse transcriptase-nested polymerase chain reaction (RT-nested PCR).

**Patients and Methods:**

From November 2007 to March 2013, 45 patients from Liver Transplant Center of Imam Khomeini Hospital, Tehran, were enrolled in this cross sectional study. PBMC specimens were separated from the peripheral blood sample. After extraction of RNA from plasma and PBMC specimens, HCV-RNA status was tested by RT-nested PCR. The 5′-untranslated region (5′-UTR) genotyping of HCV-RNA amplified from PBMC specimens was performed by a standard methodology with the INNO-LiPA^TM^ HCV II kit. The PCR products of 5′-UTR were sequenced after cloning into the pJET1.2 / blunt cloning vector.

**Results:**

Of 45 patients, 4 (8.9% [95% CI: 4.4-15.6]) had detectable genomic HCV-RNA in their PBMC specimens. HCV genotypes were determined in the PBMCs of these subjects showed that 2 (50.0%) subjects with occult HCV infection had HCV subtype 3a, and 2 (50.0%) had HCV subtype 1b.

**Conclusions:**

This study found that 8.9 % of the Iranian candidates for liver transplant with cryptogenic cirrhosis had occult HCV infection. Therefore, designing prospective studies focusing on the diagnosis of occult HCV infection in these subjects prior to liver transplantation could be valuable.

## 1. Background

Cirrhosis of the liver determined as a chronic, progressive, and degenerative disease, described by structurally abnormal nodules and fibrosis in the liver (1). Liver cirrhosis usually identified as cryptogenic cirrhosis, a number of possible recognizable etiologies must be first excluded such as viral hepatitis, alcohol abuse, autoimmune hepatitis, nonalcoholic steatohepatitis (NASH), Wilson’s disease, biliary tract disease, hepatotoxic drug, thyroid dysfunction, decompensated diabetes, haemochromatosis, any severe systemic disease, etc. The frequency of cryptogenic hepatitis is reported to be 5.4% (2). Approximately, 3-30% of patients with cirrhosishave cryptogenic cirrhosis (3-5), and its prevalence is reported to vary from 3-14% in adults to 22% in children (2). This disease is the fourth indication for liver transplantation and about 7-14 % of the recipients receive transplants for this etiology (6, 7). The diagnosis of cryptogenic cirrhosis has significantly decreased, following the discovery of viral hepatitis (8). Cryptogenic cirrhosis or cirrhosis of unknown etiology is probably a representation of the endpoint of several different occult hepatic disorders. It is an important clinical entity as patients with cryptogenic cirrhosis can develop hepatocellular carcinoma (HCC) (9). Many studies have been conducted to find an etiology for cryptogenic liver disease and recently, the importance of hepatitis C infection as a cause of liver disease with unknown etiology and hepatocellular carcinoma (HCC) has been discussed, thus, it is important to clarify the role of infection with this virus in cirrhosis with unknown etiology.

Hepatitis C virus is an important pathogen which chronically infects nearly 2.2 % of the world population (10). Iran has low endemicity for HCV infection and less than 0.2% of the general populations are infected with HCV (11). In about 85% of the cases chronic HCV infection is established. Chronic hepatitis C progresses to cirrhosis in up to 35% of the patients and approximately 3% of these patients would eventually develop HCC (12). In January 2004, a new entity of HCV infection, which was called occult HCV infection, was described in patients with cryptogenic hepatitis (13). Occult HCV infection, characterized as the presence of genomic HCV RNA strand in liver biopsy and peripheral blood mononuclear cell (PBMC) specimens in the absence of detectable level of HCV RNA in plasma by current laboratory methods, and in the absence or presence of anti HCV antibodies. This occult infection has been reported in individuals with or without chronic liver disease with unknown etiology, in several at risk groups for HCV infection, and also in general population without any evidence of liver disease (13, 14). Hepatitis C virus is essentially hepatotropic, and hepatocytes are the main site for HCV replication. The intermediary of replication of this virus is a negative-strand RNA. There is some evidence of the presence of negative chain HCV RNA in PBMCs which is not detected in plasma. Furthermore, the virus multiplying has been demonstrated in these cells of individuals with occult HCV infection (15).

End-stage liver disease (ESLD) secondary to cryptogenic cirrhosis is one of the most important indications for liver transplantation (LT) (7). The liver biopsy always plays a critical role in the management of individuals with a diagnostic difficult situation for instance the patients with abnormal liver tests with unknown etiology or having a specific liver disease (4). Although the best and most reliable technique to diagnosis occult HCV infection is testing of genomic HCV RNA strand in the liver biopsy specimens, detection of HCV RNA in PBMC specimens is a valuable method when the liver biopsy could not be performed (16).

## 2. Objectives

The main purpose of the present study was to detect the presence of genomic HCV RNA strand in PBMC specimens of the liver transplant Iranian candidates with cryptogenic cirrhosis.

## 3. Patients and Methods

### 3.1. Study Population

Forty five consecutive individuals with established cirrhosis with unknown etiology under observe of the liver transplant center of Imam Khomeini Hospital affiliated with Tehran University of Medical Sciences, Tehran Iran, were investigated from November 2007 to March 2013 in this cross sectional study by using Census method. The diagnosis of liver cirrhosis with unknown etiology was made after a comprehensive evaluation failed to identify a specific etiology. After explaining the study protocol, a written consent form was obtained from each patient. The present study was approved by the local ethics committee of Iran University of Medical Sciences.

Patients with established cryptogenic cirrhosis were screened for inclusion in the present study. The severity of the patients liver disease was scored according to the Model for End stage of Liver Disease (MELD), and the Child-Pugh classification. The inclusion criteria were as follows: 1) ultrasound showing a diffusely echogenic liver; 2) having normal or elevated ALT (more than 1.5 times); 3) all the patients had negative results for serum genomic HCV RNA and anti-HCV (tested at least three times before the entry to the study);4) Patients exclusion criteria were all known causes of cirrhosis; for instance: alcohol abuse (> 20 g/day), infection with hepatitis C virus (plasma HCV RNA and anti-HCV positive), infection with hepatitis B virus [plasma HBV DNA, hepatitis B surface antigen (HBsAg), hepatitis B e antigen (HBeAg), hepatitis B core antibody (HBcAb), and hepatitis B e antibody (HBeAb) positive], autoimmune hepatitis, genetic disorders, Wilson's disease, drug toxicity, NASH, haemochromatosis, biliary obstruction and primary biliary cirrhosis, presence of any severe systemic illness, malignancies, and positive results for anti-human immunodeficiency virus antibodies (anti-HIVAbs).

### 3.2. Collection and Preparation of the Samples

A peripheral blood sample from each patient was collected in an EDTA-containing sterile tube. After separation of plasma by centrifugation, it was stored at –80°C for experiments. The PBMCs of the samples were isolated by Ficoll Hypaque (FH) gradient centrifugation (Lympholyte-H, Cedarlane, Canada). The pellet of PBMCs was washed for more than three times using phosphate-buffered saline (PH = 7.3 ± 0.1). The cells were counted and after adding RNALater (Ambion Inc., Austin, TX) solution, were stored at –80°C until use. Plasma and PBMC specimens from 10 consecutive blood donors as well as 10 patients with established chronic HCV infection were used as negative and positive controls, respectively. Also, the patients who had an occult HCV infection undertook two extra peripheral blood samplings every three months for following up the patients.

### 3.3. Isolation of RNA and Detection of the Genomic HCV RNA Using Reverse Transcriptase-Nested Polymerase Chain Reaction (RT-nested PCR) Method

**Figure 1.**
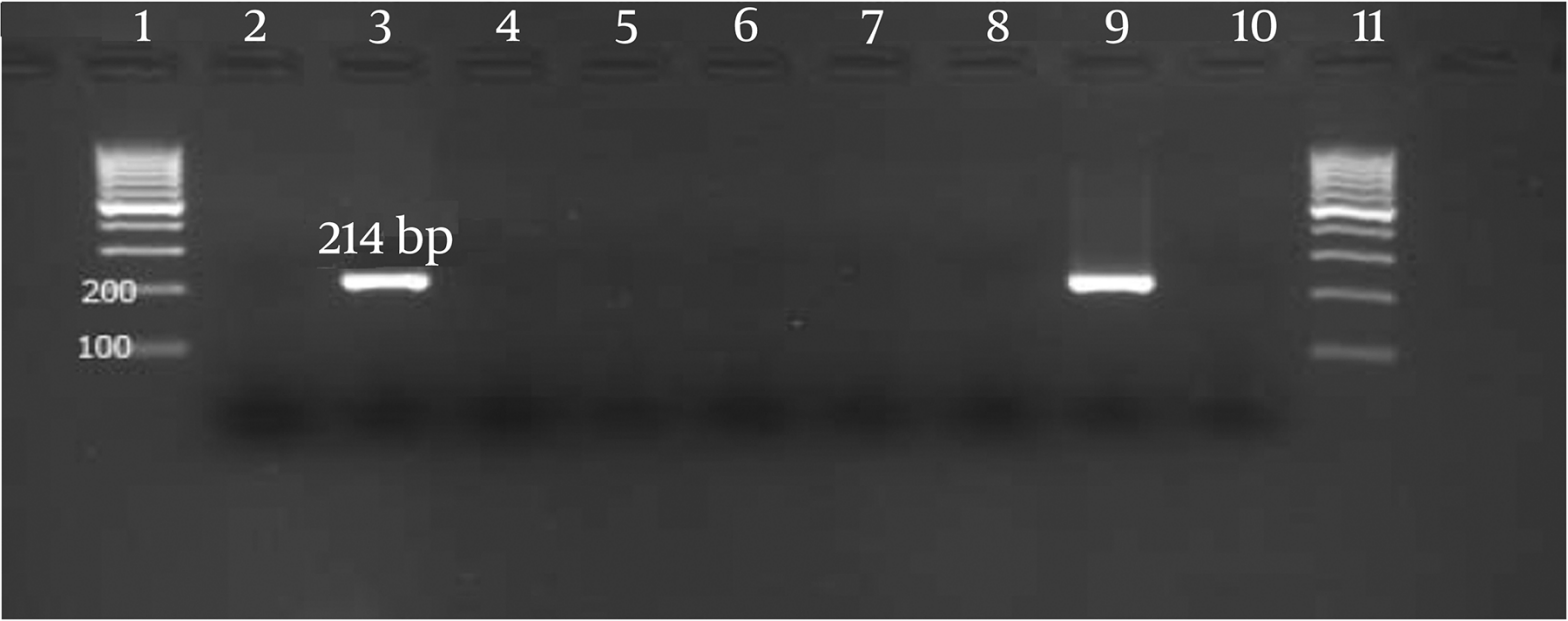
Result of HCV RNA Detection by RT Nested-PCR Method Lane1: 100 bp DNA ladder, Lane 2: PCR products of first patient's serum, Lane 3: PCR products of first patient's PBMCs, Lane 4: PCR products of second patient's serum, Lane 5: PCR products of second patient's PBMCs, Lane 6: Negative control in RT round PCR, Lane 7: Negative control in first round PCR, Lane 8: Negative control in second round PCR, Lane 9: HCV RNA positive control, Lane 10: HCV RNA negative control, Lane 11: 100 bp DNA ladder.

RNA was extracted from plasma and a pellet of about 3-5 × 10^6^ PBMC specimens using High Pure Viral Nucleic Acid Kit (Roche Diagnostics GmbH, Mannheim, Germany) according to the manufacturer’s instructions. Genomic HCV RNA in plasma and PBMC specimens was detected by RT-nested PCR method. cDNA synthesis from RNA and two-stage PCR were performed as previously described in detail (17). The PCR products of specimens with appropriate controls and the DNA size marker (100 bp) were visualized on 1.5% agarose gel stained with the fluorescent dye ethidium bromide followed by electrophoresis (Figure 1). 

The sensitivity limit of the PCR amplification method for detection of genomic HCV RNA strand was 40 IU/ml plasma. The sensitivity was determined by testing serial dilutions of a plasma specimen with a known viral load [determined using Cobas TaqMan 48 Analyzer (Roche, Germany)].

### 3.4. HCV Genotyping by INNO-LiPA

**Figure 2.**
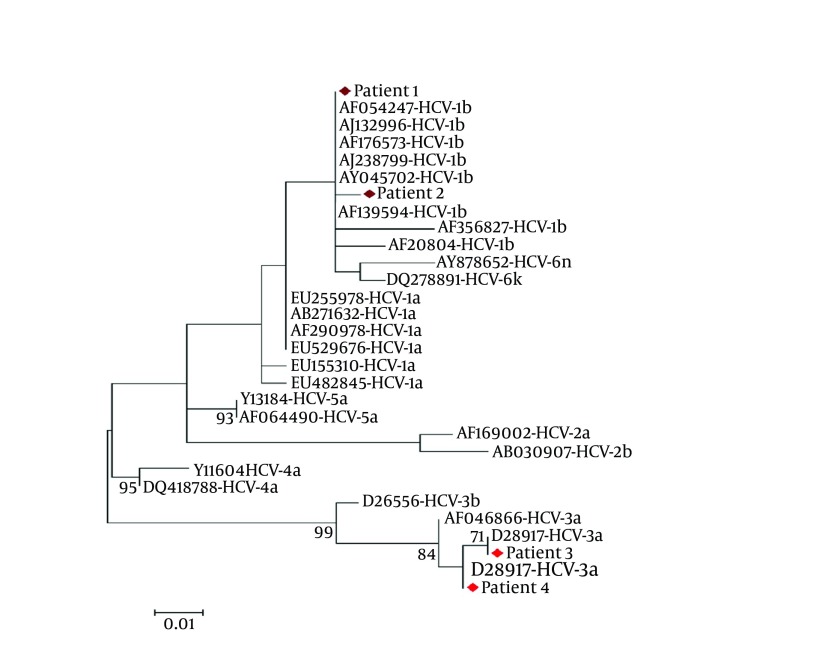
Neighbor-joining tree constructed with HCV 5′-untranslated region (5′-UTR) nucleotide sequences of the clones obtained from 4 individuals with occult HCV infection, and sequences corresponding to different HCV genotypes retrieved from GenBank. Bootstrap values ≥70 obtained after 1000 replicates of the data sheet, has been shown in the nodes of the tree.

Total RNA was extracted from plasma and PBMC specimens as described above. The 5′-UTR genotyping of HCV RNA amplified from PBMC specimens was performed by the INNO-LiPA^TM^ HCV II kit (Innogenetics, Ghent, Belgium) according to the manufacturer’s instructions. This HCV genotyping was also confirmed by sequencing 5′-UTR fragments after the PCR products were cloned into the pJET1.2/blunt cloning vector (Fermentas, St. Leon-Rot, Germany) as described elsewhere (17, 18) (Figure 2).

### 3.5. Enzyme Immunoassay (EIA) and Western Blot Assay for Detection of Anti-HCV Antibodies

Anti-HCV antibodies were determined by two different commercial enzyme-linked immunosorbent assay (ELISA) kits: a forth generations test [INNOTEST-HCVAb IV (Innogenetics, Ghent, Belgium)], and a third generation test [ACON Laboratories, San Diego, CA]. Western blot assay was performed using HCV BLOT 3.0 (MP Diagnostics, Germany) according to the manufacturer’s instructions.

### 3.6. Statistical Analysis

The statistical analyses were performed by Chi Square and Fisher’s exact tests . Analysis of continuous variables was performed using Student’s t-test and Mann-Whitney U. The Kolmogorov-Smirnov test was used for checking the normal distribution of the data. The descriptive statistical indexes including standard deviation, mean, confidence interval at 95%. P value of ≤0.05 was considered to be statistically significant. All data were analyzed using SPSS version 17 (SPSS, Chicago, IL, USA).

## 4. Results

Forty five individuals with established cryptogenic cirrhosis, whose results for plasma HCV RNA strand and anti-HCV antibodies testing were repeatedly negative, were recruited in this cross sectional study. The mean age of participants was 41.4 ± 12.4 (range 19-64 years). Of 45 patients, 33 (73.3%) were male. All patients had negative findings for antibodies against HCV and genomic HCV RNA in their plasma specimen. The complete information of the participants with established cryptogenic cirrhosis is summarized in Table 1. 

**Table 1.** Characteristics of the Patients With Established Cryptogenic Cirrhosis

Characteristics	Total	Negative	Positive	P value
**No.**	45	41	4	
**Gender Male/Female**	33/12	30/11	3/1	0.917^a^
**Age, y**	41.7 ± 12.3 (19- 64)	40.8 ± 12.5	50.8 ± 5.8	0.019^b^
**Body-Mass-Index (BMI), kg/m2**	24.2 ± 5.6 (16.9- 39.1)	24.2 ± 5.8	23.9 ± 2.3	0.196^b^
**Laboratory Parameters, Mean ± SD**				
White Blood Cell	4252.0 ± 1979.6 (1500-9600)	4179.8 ± 1819.0	4975.0 ± 3519.8	0.036^b^
Hemoglobin, gr/dl	12.2 ± 1.8 (8.4-15.5)	12.3 ± 1.9	11.0 ± 3.0	0.080^b^
Alanine aminotransferase, IU/L	48.6 ± 38.4 (10.0-210.0)	42.9 ± 24.0	105.8 ± 94.3	<0.001^b^
Aspartate aminotransferase, IU/L	65.6 ± 60.8 (4.8-380.0)	61.1 ± 56.6	110.8 ± 91.0	0.331^b^
alkaline phosphatase, IU/L	309.3 ± 149.6 (74.0-770.0)	301.7 ± 142.5	385.50 ± 220.0	0.317^b^
Bilirubin Total, mg/dl	2.8 ± 2.8 (0.6-17.2)	2.8 ± 3.0	2.7 ± 1.2	0.595^b^
Bilirubin Direct, mg/dl	0.8 ± 1.1 (0.1-6.9)	0.9 ± 1.1	0.6 ± 0.3	0.829^b^
Cholesterol, mg/dl	161.3 ± 34.4 (103.0-241.0)	160.3 ± 32.3	171.8 ± 57.0	0.095^b^
Triglyceride, mg/dl	107.1 ± 39.4 (45.0-211.0)	108.5±40.2	93.5 ± 32.7	0.667^b^
Iron, mg/dl	76.1 ± 51.5 (8.0-202.0	77.1±53.8	65.8 ± 14.5	0.031^b^
Ferritin, mg/dl	87.9 ± 141.3 (5.0-750.0)	73.0 ± 101.7	237.8 ± 345.0	0.239^b^
Platelet	78431.8 ± 65373.8 (12000-352000)	81825.0 ± 66935.0	44500.0 ± 36391.4	0.604^b^
Prothrombin Time, Sec	17.0 ± 3.2 (12.0-26.7)	17.1 ± 3.3	15.8 ± 2.4	0.423^b^
INR	1.7 ± 0.6 (1.0-3.6)	1.8 ± 0.6	1.5 ±0.4	0.366^b^
**Epidemiological Parameters, No. (%)**				
History of Surgery	33 (73.3)	30 (73.2)	3 (75.0)	0.938^a^
History of dental operation	28 (62.2)	26 (63.4)	2 (50.0)	0.601^a^
History of jaundice	6 (13.3)	5 (12.2)	1 (25.0)	0.465^a^
History of blood transfusion	21 (46.7)	17 (41.5)	4 (100.0)	0.027^a^
History of endoscopy	44 (97.8)	40 (97.6)	4 (100.0)	0.754^a^
History of war injury	4 (8.9)	3 (7.3)	1 (25.0)	0.241^a^
Hospital admission	42 (93.3)	29 (70.1)	4 (100.0)	0.581^a^
History of travel to the endemic area	9 (20.0)	5 (12.2)	4 (100.0)	<0.001^a^

^a^P value base on Fisher Exact test

^b^P value base on Mann-Whitney U test

The genomic HCV RNA was detected in PBMCs from 4 (8.9 %) of the 45 patients who have cirrhosis with unknown etiology. Therefore, these patients had an occult HCV infection. For confirmation of positive results, these patients undertook two extra peripheral blood samplings every three months. The presence of genomic HCV RNA strand in plasma and PBMC specimens was tested again in these patients, which the Preliminary results were confirmed. The HCV genotyping was performed by the INNO-LiPA^TM^ HCV II kit in PBMC specimens of the patients with occult HCV infection showed that 2 (50%) patients were infected with HCV subtype 3a, and 2 (50%) with HCV subtype 1b. The results of the HCV genotyping of the patients with occult HCV infection using the INNO- LiPA^TM^ HCV II kit was confirmed by the nucleotide sequence analysis of the HCV 5′-UTR.

The plasma of all the individuals with occult HCV infection was examined twice for detection of anti-HCV Abs with two different commercial kits. None of them had positive results for anti-HCV Abs in their plasma. The presence of anti-HCV Abs was tested in the individuals’ plasma with occult HCV infection by a third-generation recombinant immunoblot assay; one of 4 individuals with occult HCV infection had anti-HCV Abs to NS3-2 antigen and therefore, the results of western blot assay in these patients were indeterminate.

In this cross sectional study, significant differences were seen in the age (P= 0.02), white blood cell count (P= 0.04), alanine aminotransferase (P<0.001), iron (P= 0.03), history of blood transfusion (P= 0.03), and history of travel to the endemic area (P<0.001) between patients with positive and negative results for occult HCV infection (Table 1). 

## 5. Discussion

The recurrence of the HCV infection is one of the most important problems following liver transplantation which leads to a decrease in patient and graft failure (19). Therefore, identification of patients with HCV infection for treatment planning before and after liver transplantation is critical. Newly, occult HCV infection has been characterized as the presence of genomic HCV RNA in liver biopsy specimen and in nearly 70% of the cases in PBMC specimens despite undetectable genomic HCV RNA as well as antibodies against HCV in the plasma (13). The current study was primarily aimed at determining the presence of HCV RNA in PBMC specimens of the patients with cryptogenic cirrhosis who were candidates for liver transplant. The genomic HCV RNA was detected in PBMCs of 4 (8.9%) of 45 patients, therefore, these patients had occult HCV infection. The HCV genotyping of HCV RNA detected in PBMCs of individuals with occult HCV infection showed that 2 (50.0%) patients were infected with HCV subtype 3a, and 2 (50.0%) with HCV subtype 1b. Despite the fact that occult HCV infection has been recently found, this infection has been reported from various parts of the world for instance: it was seen in individuals with cryptogenic liver disease in Spain (57%) (13), in Egypt (10%) (20), in Pakistan (74%) (21), In Iran (10.1%) (17), in individuals with Cryptogenic cirrhosis with HCC in Italy (40%) (22), in haemodialysis patients in (45%) in Spain (16), patients with lymphoproliferative disorders in Iran (1.9%) (18), in the general population (3.3%) in Italy (23), in population free of clinically detectable infectious liver disease (1.27%), and in patients with active HBV infection (28%) in Italy (24). However, there are also several reports which scientists have not been able to trace the occult HCV infection, such as in mixed cryoglobulinemia (25), non-Hodgkin lymphoma (26), and autoimmune disorders (27). So it seems that more studies in this field on various groups with high population size are needed.

Occult HCV infection is distributed all over the world and it seems that all of the HCV genotypes are involved in this infection. In the preliminary studies on the occult HCV infection the only HCV subtype isolated was 1b (13, 16). However, subsequent studies have revealed occult HCV infection belonging to HCV subtypes 1a, 1b, 2a, 3a, 3b, 6f (14, 17, 21). The most prevalent HCV genotypes circulating throughout Iran are 1a (44.9%) followed by subtype 3a (39.6%), and 1b (11.3%) (28). We detected only HCV subtypes 3a and 1b in our study population, despite the most abundant HCV genotype in Iran is 1a. Thus it seems that more studies in this field are needed with large population size. On the other hand, it has been reported that different HCV genotypes are detected in PBMC specimens as compared to plasma of the patients with occult HCV infection, or HCV infection (15, 23). The prevalence rate of occult HCV infection in the present study (8.9%) was compatible with the prevalence rate of occult HCV infection in Iranian patients (10.1%) with cryptogenic liver disease (17) and it is several times of the prevalence of HCV infection in general population (0.2%) of Iran (11). Thus it seems that this type of infection (occult HCV infection) should be considered, and blood transfusion may never be completely risk-free. Although screening for infectious diseases has significantly improved the safety of blood transfusion, the risks of transfusion of different blood borne diseases (such as hepatitis C and B, HIV/AIDS, and etc.) have not been eliminated (23). The risk of HCV infection has considerably reduced because of more sensitive and reliable nucleic acid testing (29); however, due to their costs, they are not widely used in developing countries. These countries routinely check for antibodies to this virus; thus, the test cannot recognize HCV infection that occurs among the time of exposure to the infection and emergence of antibodies to the virus (known as the window period) (23, 29).

There are different risk factors for HCV infection such as transfusion history, unsafe injections, tattooing, intravenous drug abuse, razor blade shaving by barbers, and extramarital sexual contacts. There are a lot of studies demonstrating that contact with contaminated blood and other body fluids occur in a variety of occupations. Health care workers and public safety personnel can be exposed to blood through needle sticking and other sharps injuries, and skin exposures. One of the most important pathogens that can infect humans through these ways is HCV (30, 31). Interestingly, significant differences were seen in a history of blood transfusion, and history of travel to the endemic area among individuals with and without occult HCV infection. Thus, this infection may be a result of transmission from contaminated blood or contact of an injured tissue with blood or body fluids. Also a significant difference was observed in the age between individuals with and without occult HCV infection. This is probably due to exposure to the infection by various pathogens with increasing the age. It has been reported that there is a potential transmission risk of occult HCV infection because of finding high occult HCV infection frequency between family members of occult HCV positive patients (32). This is likely due to the presence of the virus in peripheral blood mononuclear cells of patients with occult HCV infection. So the likelihood of transmission of the HCV infection in the blood transfusion and bone marrow and organ transplantation is considered. It was also reported that nearly 20% of individuals with positive results for HCV undergoing liver transplant likely develop liver cirrhosis in up to 5 years, and in about 50% of cases probably develop liver cirrhosis within a decade (19). Therefore, identification of occult HCV infection and HCV infection before liver transplantation for patients and physicians has particular importance. Also, the reactivation of HCV infection is well known in immunocompromised patients or individuals receiving immunosuppressive therapy (33). There is a case report that revealed occult HCV infection may play a critical role as an agent of liver failure in transplanted patients (34). Thus, the risk of HCV transmission should be considered in liver transplantation and it seems that performing the test to detect infection prior to liver transplantation is important and necessary.

Due to the lack of antibodies to the HCV in plasma of patients with occult HCV infection detection of antibodies against HCV is not distinguishing. In the present study, we tested plasma samples for detection of anti-HCV Abs with two commercial enzyme immunoassay kits. The results showed that all 45 patient's plasma samples which were tested for anti-HCV Abs had negative results ; thus, the results were compatible with previous reports (35). All 4 plasma specimens of patients with occult HCV infection were also examined for detection of anti-HCV Abs by a third generation recombinant immunoblot assay (RIBA). This test has been used for confirming samples with positive findings for HCV antibodies by enzyme immunoassay and positive results of RIBA for detection of anti-HCV Abs is usually related to HCV viremia (36). In the present study, one (25.0%) of 4 patients with occult HCV infection had anti-HCV Abs to NS3-2 antigen. However, this patient had antibody reacting to a single recombinant HCV antigen and therefore the result was determined as indeterminate. This finding could be important, but further research is required. In the present study we tested only 45 patients, because the number of these patients is too low, and finding them is time consuming. Thus, according to the results of the present study, the occult HCV infection should be considered in the liver transplant candidates with cryptogenic cirrhosis, and it seems that a multicenter study with a larger sample size is necessary to confirm the presence of this infection.

In conclusion, the present study demonstrated that more than 8.9% of liver transplant candidates with cryptogenic cirrhosis had occult HCV infection. So, HCV RNA detection is recommended in PBMC samples of the individuals with cryptogenic cirrhosis prior to liver transplantation especially in patients who are suspected to occult HCV infection. Finally, future studies should be performed to consider the possibility of this infection in various groups of the community who have been associated with the infectious agents transmitted through blood.
